# Rectal Diverticula as a Causative Factor in Pelvic Inflammation in Women

**Published:** 1917-07

**Authors:** Walter C. Swayne

**Affiliations:** Obstetric Physician, Bristol Royal Infirmary, and Professor of Obstetrics, University of Bristol


### RECTAL DIVERTICULA AS A CAUSATIVE FACTOR IN PELVIC INFLAMMATION IN WOMEN.

BY

WALTER C. SWAYNE, M.D. Lond. and Bristol,

*Obstetric Physician, Bristol Royal Infirmary, and Professor of Obstetrics, University of Bristol.*

THE importance of diverticula of the lower part of the large intestine as a factor in the production of general or localised peritonitis was once again emphasised when in 1908

Maxwell Telling published a paper[1] giving an account of over a hundred cases, the records of which he had collected. The great majority of Telling's cases dealt with diverticula of the sigmoid and descending colon ; but in the course of his paper he drew attention to the fact that the same effects were produced by diverticula of that part of the rectum which is covered with peritoneum.

In 1910 Cameron and Rippmann[2] published a paper, giving an account of the post-mortem findings in five cases of death from general peritonitis or localised inflammation in which, at the autopsy, no lesion could be found excepting a perforated or inflamed diverticulum.

Telling points out that the following affections may be caused by diverticula :—

1. General infective peritonitis from perforation.

2. Acute or gangrenous inflammation (acute diverticulitis).

3. Chronic proliferative inflammation with stenosis of the intestine.

4. Adhesions, especially to (*a*) small intestine, (*b*) bladder.

5. Perforation with (*a*) general peritonitis, (*b*) local abscess, (*c*) sub-mucous fistula, (*d*) fistula into bladder or other hollow viscera.

6. Impaction of foreign bodies, *e.g.* a fishbone (as in a case recorded by Cameron).

7. Inflammation of the sigmoid mesentery.

8. Metastatic suppuration.

9. Local peritonitis.

10. Development of malignant disease.

11. Perforation into a hernial sac.

In several of the cases recorded by Telling and Cameron and Rippmann the symptoms were attributed to appendicitis, but in no instance was any abnormality of this structure found.

[1] *Lancet*, March 21st. [2] Guy's Hospital Reports.

As these diverticula are actual hernial openings in the wall of the intestine at the points of attachment of the appendices epiploicæ with protrusion of the mucous membrane, and as they occur for the most part in the lower part of the intestine, and very commonly contain concretions of solid fæcal matter, the risks which they cause hardly need explanation.

The relative frequency of the different occurrences mentioned by Telling are approximately as follows :—

In Telling's paper rather more than one-third of the cases dealt with resulted in perforation, a rather larger number resulted in the formation of more or less dense adhesions. Of the remaining cases, about equal numbers suffered from one or other of the affections mentioned in his list.

Practically all the results which Telling and Cameron have observed in cases of diverticula of the sigmoid can occur in cases where diverticula are situated in the upper part of the rectum.

In addition to the pathological conditions already described, mistakes in diagnosis may be caused by the presence of diverticula when acute or sub-acute symptoms are absent altogether, *e.g.* the nodular sensation imparted to the examining finger may lead to their being mistaken for a new growth possibly of a malignant nature.

The first case which I am about to describe is an example of such a mistake in diagnosis, the second of my cases is one in which perforation occurred with the formation of a fistulous opening into the bladder.

These diverticula are seldom found in women below middle age, but are fairly common in the aged. No treatment is likely to be successful ; medicine does not affect them, and their operative removal, seeing that they are generally multiple, means a tedious, difficult, and dangerous operation.

**Case 1.**—A multipara, aged 51, was sent to me for diagnosis by her own medical man, who was of opinion that she had a malignant growth of the intraperitoneal portion of the rectum. As I was unable to give a definite opinion without a laparotomy, the abdomen was opened in the middle line, and the nodules felt on vaginal and rectal examination examined. These were seen to be diverticula occurring in the appendices epiploicæ of the rectum, which contained hard nodules. One was removed for the purpose of more detailed examination. Its stalk was ligatured, and inverted into the rectum by means of a purse-string suture. On cutting into the appendix epiploica, it was found to contain a hard concretion of fæcal matter, and the nature of the nodules sufficiently demonstrated.

**Case 2** was an unmarried woman, aged 56, who had consulted me at intervals during the previous ten years on account of a fibroid growing from the posterior wall of the uterus, presenting the usual symptoms, but for which operative treatment was not advised, as the growth did not enlarge, and latterly showed definite signs of diminishing. She now consulted me on account of pain in the region of the bladder and frequent micturition, with the added complaint that she not infrequently passed flatus through the urethra.

The urine contained pus, was extremely offensive, and gave a plentiful growth of bacillus coli ; minute pieces of fæcal matter were occasionally, but not frequently, found.

On examination a mass was felt on the posterior wall of the uterus, which was supposed to be the remains of the fibroid, much diminished in size. A diagnosis was made of an adherent rectal diverticulum, which had perforated into the bladder. As local treatment of the bladder only partially ameliorated her symptoms, and was ineffectual in producing permanent cure, it was decided to deal with the condition after laparotomy. When the abdomen was opened, it was found that a rectal diverticulum was densely adherent to the fundus of the bladder above the fundus of the uterus. This was separated, the pedicle of the diverticulum ligatured, and its stalk invaginated into the rectum by means of a purse-string suture. The perforation into the bladder was inverted by a purse-string suture, with a second row of interrupted sutures for additional security. The patient made an uneventful recovery, excepting that some suppuration occurred, and a fæcal fistula through the abdominal wall eventually developed. Her bladder symptoms cleared up almost at once, but the fistula remained. This gradually contracted until, although it did not completely heal, it gave her very little trouble. Owing to her condition (she was an absolute cripple from rheumatoid arthritis), it was not considered advisable to do a second operation to close the fistula. At the operation the remains of the fibroid were found to be reduced to about the size of the half of a large walnut, but covered by adhesions produced by inflammation of the diverticulum, which had made its size appear very much larger.

The points on which the diagnosis rested were these: it was obvious that there was a communication between the bladder and the intestine; it also appeared obvious that this connection was not with a part of the intestine, of which the contents are fluid under normal conditions. A communication with the small intestine possibly through a persistent diverticulum of Meckel was thus excluded. The possibility of a communication between the appendix and the bladder was considered, but here again fluid fæcal matter would have been present and probably persistent, and the patient had at no time presented symptoms which suggested any inflammation of that structure. The lower part of the large intestine, therefore, seemed to be the most probable site of the communication, and it was suggested that this was possibly due to an inflamed adherent diverticulum, which at the operation proved to be the case.

Other cases which I am unable to cite in detail have come under my notice. In one the opening of a pelvic abscess containing fæcal smelling pus resulted in a fæcal fistula both through the vagina and abdominal wall. In another the opening in the hypogastrium of a superficial abscess of the abdominal wall revealed a track leading into the pelvis. In this case also a fæcal fistula from the abdominal opening impinged on the rectal wall below the promontory of the sacrum, easily felt by the finger when strong pressure was made upwards, in the posterior fornix of the vagina. In both of these cases the origin of the suppuration was probably an inflamed diverticulum.

That the presence of these diverticula may be the cause of inflammation in the pelvis should not be forgotten, nor the fact that they may present appearances similar to those of malignant disease.

